# PeachDB: accelerating breeding by reducing cost with AI-powered genomic selection in peach

**DOI:** 10.1093/hr/uhag040

**Published:** 2026-02-16

**Authors:** Qingyuan Han, Yuhao Lou, Tianyu Zhu, Hongzhao Jin, Lixuan Zhang, Bo Zhang

**Affiliations:** Zhejiang Key Laboratory of Horticultural Crop Quality Improvement, Department of Horticulture, Zhejiang University, Zijingang Campus, Hangzhou 310058, China; Zhejiang Key Laboratory of Horticultural Crop Quality Improvement, Department of Horticulture, Zhejiang University, Zijingang Campus, Hangzhou 310058, China; Zhejiang Key Laboratory of Horticultural Crop Quality Improvement, Department of Horticulture, Zhejiang University, Zijingang Campus, Hangzhou 310058, China; Zhejiang Key Laboratory of Horticultural Crop Quality Improvement, Department of Horticulture, Zhejiang University, Zijingang Campus, Hangzhou 310058, China; Zhejiang Key Laboratory of Horticultural Crop Quality Improvement, Department of Horticulture, Zhejiang University, Zijingang Campus, Hangzhou 310058, China; Zhejiang Key Laboratory of Horticultural Crop Quality Improvement, Department of Horticulture, Zhejiang University, Zijingang Campus, Hangzhou 310058, China; Hainan Institute of Zhejiang University, Sanya, Hainan 572000, China

Dear Editor,

The unprecedented expansion of multiomics datasets is driving a profound paradigm shift in life science research. However, the effective reutilization of these resources by breeders—particularly those without extensive bioinformatics expertise—remains hampered by data fragmentation and a lack of standardized analytical framework. While comprehensive plant databases such as PlantGIR and TVIR2 have proliferated recently [1, 2], there remains a conspicuous shortage of deeply integrated, species-specific platforms designed to directly bridge the gap between high-throughput data and practical horticultural breeding. The peach is a globally cultivated economic crop and serves as a model species for the Rosaceae family. Unlike annual field crops, the peach is a perennial woody fruit tree, which presents two major challenges in breeding programs. First, the juvenile phase can last up to three or more years, during which flowering and fruiting do not occur, thereby extending the breeding cycle. Second, each peach tree requires considerable growing space, significantly more than herbaceous crops such as tomatoes and rice, thereby leading to high land and operational costs. These constraints underscore the need for early selection technologies that can reduce resource input and accelerate breeding efficiency. To address these challenges, we developed Peach Database (PeachDB http://peachdb.cn/), a multiomics platform for peach, and implemented an artificial intelligence (AI)-driven genomic selection model. This approach enables accurate prediction of fruit quality traits at the seedling stage, effectively shortening the breeding cycle, reducing land utilization by 86%, and substantially lowering breeding costs.

The quality attributes of peach fruit are diverse, with flavor being the most complex trait, comprising soluble sugars, organic acids, and aromatic compound—characteristics that exhibit intricate quantitative variation. We collected data on 41 chemical components and their concentrations relevant to peach fruit quality, including malic acid, citric acid, quinic acid, fructose, glucose, sucrose, and sorbitol (Fig. 1A). Genomic sequences from 21 peach types, encompassing both wild and cultivated species, specifically *Prunus persica, P. davidiana, P. kansuensis, P. ferganensis*, and *P. mira*, were analyzed, covering approximately 15 000 000 site variants. In parallel, we compiled re-sequencing data from 1456 samples across five global subgroups (Fig. 1A). The PeachDB database integrates widely used bioinformatics tools such as JBrowse and BLAST, enabling efficient genome navigating and sequence comparison PeachDB offers 246 Genome Selection (GS) models to facilitate peach breeding programs. Users can download the appropriate model from PeachDB, refer to the accompanying user manual, and input genetic variation data to predict the phenotypic traits of the target population.

**Figure 1:**
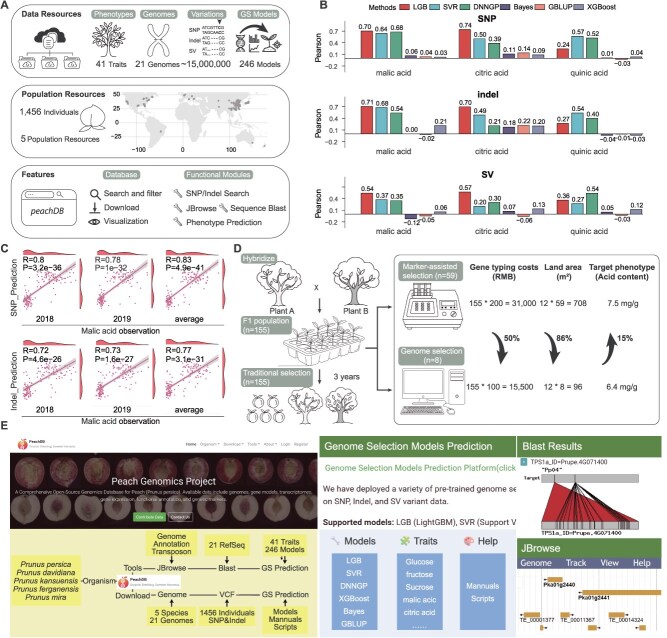
Overview of the Peach Database. (A) Data resources, population resources, and a summary of key features and functional modules in PeachDB. (B) Genomic selection model prediction accuracy on single-nucleotide polymorphisms (SNPs), insertion or deletion (Indels), and structural variants (SVs). LGB, light gradient boosting machine; SVR, support vector regression; DNNGP, deep neural network for genomic prediction; Bayes, Bayesian LASSO; GBLUP, genomic best linear unbiased prediction; XGBoost, eXtreme Gradient Boosting. (C) Prediction accuracy of malic acid models in hybrid populations. (D) Comparative analysis of genomic selection and molecular marker-assisted breeding approaches. (E) Homepage and system architecture of PeachDB, highlighting integrated tool including the GS models and associated files, Blast tool, and JBrowse genome browser.

A natural population of peaches (*n* = 269) was used to evaluate fruit quality traits and genetic variation data, including single nucleotide polymorphisms (SNPs), insertions/deletions (Indels), and structural variants (SVs). Six genome-wide predicted models were trained and assessed: Deep Neural Network Genomic Prediction (DNNGP), Genomic Best Linear Unbiased Prediction (GBLUP), eXtreme Gradient Boosting (XGBoost), Bayesian LASSO (Bayes), LightGBM (LGB), and support vector regression (SVR). Model performance was evaluated based on predictive accuracy for quality traits, quantified using the Pearson’s correlation coefficient. Compounds such as malic acid, citric acid, and quinic acid are key contributors to peach fruit flavor and play a significant role in shaping consumer preferences. The prediction accuracies for organic acids were as follows: citric acid (0.74), malic acid (0.71), and quinic acid (0.57) (Fig. 1B). Among these, SVR, LGB, and DNNGP demonstrate superior predictive performance. Subsequently, an artificial hybrid population (*n* = 155) was established and phenotypic data were collected over two consecutive growing seasons to assess model generalization ability. Only models with prediction accuracy exceeding 0.7 in the training population were selected for validation in the hybrid population. The citric acid model exhibited inadequate generalization capability, with the maximum prediction accuracy in the hybrid population reaching merely 0.22. In contrast, the malic acid prediction model exhibited strong generalization performance, with all evaluated models achieving prediction accuracies above 0.7, and the highest accuracy reaching 0.83 (Fig. 1C).

Subsequently, we evaluated the breeding efficiency of GS in comparison with marker-assisted selection (MAS) method. Initially, previously documented molecular markers associated with organic variation in peach fruit were utilized. Five molecular markers were correlated with organic acid content variations in peach fruit in different cultivars (*P*-value < 0.05), confirming their potential utility. These five molecular markers were then applied to a hybrid population, and among 155 hybrid materials, 59 offsprings exhibited the ‘low acid’ genotype across all five molecular markers. Building on the results from Fig. 1B, three models—Indel-LGB, SNP-LGB, and SNP-SVR—with superior predictive accuracy for malic acid, citric acid, and quinic acid, were employed to identify individuals with reduced organic acid content within the hybrid population. Based on the predictions from each model, hybrid materials were ranked in descending order of predicted organic acid levels. The lowest one-third of materials from each model were selected as candidates, and those consistently predicted to have low levels of all three acids were identified by intersecting the candidate sets. In 2018, the mean total acid content in the GS-selected population was 5.10 mg/g, lower than the 6.11 mg/g observed in the MAS population (a 16.5% reduction, *P*-value = 0.029). In 2019, the corresponding values were 7.71 and 8.88 mg/g, respectively (a 13.13% decrease, *P*-value = 0.138). Overall, the mean total acid content in the GS population was 6.41 mg/g, which was significantly lower than the 7.46 mg/g in the MAS population (a 14.12% reduction, *P*-value = 0.029, Fig. 1D).

The number of low-acid individuals required for retention using GS screening (*n* = 8) is 86% lower than that required with MAS (*n* = 59). In practical application, GS can reduce the genotyping cost per hybrid offspring from around 200 Chinese yuan (Renminbi, RMB) for MAS to 100 Chinese yuan, representing a 50% reduction in costs. Concurrently, the land area required for evaluation is reduced by 86%, while the phenotypic performance is improved by 15% (Fig. 1D). Therefore, GS enables not only the efficient identification of low-acid individuals with superior phenotype but also significantly reducing breeding costs and land resource requirements.

In summary, PeachDB is publicly accessible at http://peachdb.cn/ (Fig. 1E), offering downloadable GS models and genomic resources for immediate implementation in peach breeding programs. Given the rigorous demands of fruit tree breeding, our findings highlight the strong applicability of AI-driven genomic selection in peach improvement, substantially lowering operational costs and providing a valuable reference for breeding other fruit crops. While single-trait predictions are effective, a promising direction for future development is the integration of multitrait genomic selection models. Such models can leverage genetic correlations between traits to enhance the prediction accuracy of low-heritability traits, potentially incorporating multiomics data and novel algorithmic frameworks. In the future, we plan to incorporate additional genomic and phenotypic data, along with environmental variations, into model training to further improve prediction accuracy and generalization and to develop online prediction tools to extend functionality to additional traits.

## Data Availability

The data that supports the findings of this study are available in the Supporting Information and PeachDB (http://peachdb.cn/) of this article.
